# Serum indoxyl sulphate and its relation to albumin and α_1_-acid  glycoprotein as a potential biomarkers of maternal intestinal metabolism  during pregnancy and postpartum

**DOI:** 10.1371/journal.pone.0259501

**Published:** 2021-11-05

**Authors:** Barbara Lisowska-Myjak, Hanna Zborowska, Radosław Jaźwiec, Maria Karlińska, Ewa Skarżyńska

**Affiliations:** 1 Department of Biochemistry and Clinical Chemistry, Medical University of Warsaw, Warsaw, Poland; 2 Department of Laboratory Diagnostics, Medical University of Warsaw, Warsaw, Poland; 3 Institute of Biochemistry and Biophysics, Polish Academy of Sciences, Warsaw, Poland; 4 Department of Medical Informatics and Telemedicine, Medical University of Warsaw, Warsaw, Poland; University of Life Sciences in Lublin, POLAND

## Abstract

**Background:**

Serum indoxyl sulfate (IS) levels depend on the production of indole in the gut. The biological effects of IS in the vascular bed could be confirmed by changes in the levels of individual serum proteins during normal pregnancy and in the postpartum period as compared with non-pregnant controls. Albumin (Alb) and α_1_-acid glycoprotein (AGP, orosomucoid) are the most abundant serum carrier proteins with potential interrelationships with serum levels of IS.

**Methods:**

Serum levels of IS, Alb and AGP were measured in 84 pregnant women in the first, second and third trimester of pregnancy and in the postpartum period, as well as in non-pregnant controls (n = 20), using ultra-performance liquid chromatography (UPLC) coupled to mass spectrometry (IS), colorimetric assay (Alb) and immunoturbidimetric assay (AGP).

**Results:**

The postpartum serum levels [mg/L] of IS were lower (p = 0.027) than in the second trimester (mean±SD: 0.85±0.39 vs 0.58±0.32). There were no differences in the IS to ALB ratio calculated in the three trimesters of pregnancy, the postpartum period, and in the non-pregnant controls. The IS/AGP ratio increased from the first to the second trimester (p = 0.039), and decreased in the postpartum period (p<0.05), when it was lower than in the second and third trimester.

**Conclusions:**

The variability of the serum IS/AGP ratio during pregnancy and in the postpartum period may reflect shared involvement in the regulation of their intravascular relationships. The link between serum levels of IS derived from the gut and AGP could serve a potential biomarkers of maternal intestinal metabolism during pregnancy and postpartum.

## Introduction

Gut-specific serum markers measured in pregnancy and the postpartum period may be a source of information about the biological role of the gut in maintaining homeostasis of the materno-fetal complex.

Indoxyl sulfate (IS) is a small-molecular-weight endogenous metabolite (213 Da) of dietary L-tryptophan and its synthesis and serum levels depend on gut, liver and kidney function [1–6]. The colon is the main anatomical site where indole is produced by bacterial species converting dietary tryptophan into indole, which is further processed in the liver to IS and cleaved via secretion in the proximal tubules [7, 8]. Clinicians have investigated the use of IS in the diagnosis of numerous renal and non-renal diseases for more than a century, but the role of IS in the pathomechanism of disease processes remains unclear [4]. While the presence of indole in the gastrointestinal lumen has a beneficial effect by enhancing epithelial barrier function in the colon [7, 8], serum IS is an endogenous toxin, especially when it is accumulated in the serum in renal insufficiency [6, 9–11].

IS binds to serum proteins at >90%, but the degree of its binding to individual serum proteins remains unknown [12]. Albumin (Alb) and α_1_-acid glycoprotein (AGP, orosomucoid), produced in the liver, are the most important serum carrier proteins that play a role in transporting endogenous and exogenous ligands [13, 14]. The two proteins differ in their serum levels, properties and biological functions. Alb is the most abundant human serum protein, a negative acute phase protein [13]. AGP is a positive acute phase protein, which plays an important role in inflammation and regulation of immune responses. A few studies have confirmed a relationship between AGP and mucosal inflammatory changes in the intestine [14–17]. Understanding of the mechanisms underlying the regulation of serum levels of IS and individual proteins, and hence their effectiveness in joint performance of biological tasks, could facilitate selecting a specific biomarkers to evaluate physiological and pathological processes in which they are involved.

Currently, there are very few intestine-specific laboratory parameters that are easily available and non-invasive, and could be used to differentiate between numerous alterations in the anatomy and physiology of the gastrointestinal tract during pregnancy and gastrointestinal disorders in pregnant patients [18, 19].

The aim of the present study was to assess the relationships between serum IS, Alb and AGP levels in each trimester of normal pregnancy and the postpartum period *versus* the non-pregnant state, and to explore the possible correlations between these values.

## Material and methods

### Ethics statement

This study was approved by the Medical Ethics Committee at the Central Clinical Hospital of the Ministry of the Interior and Administration, Warsaw, in accordance with the Declaration of Helsinki, Decision No 71/2011. The women participating in the study (pregnant and non-pregnant women) signed statements confirming the informed consent for participating in the study.

### Subjects

**Fig 1:**
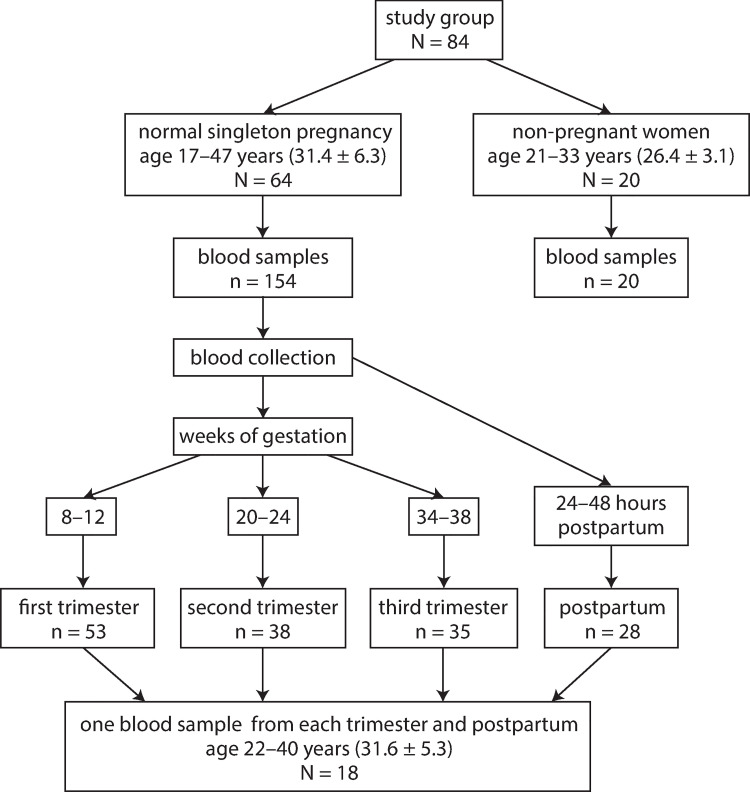
Study design: Blood sample collection scheme in pregnant, postpartum, and non-pregnant women.

The study used blood samples from 84 pregnant women and 20 non-pregnant women. According to routine recommendations for prevention and treatment of maternal peripartum infection, microbiological tests for GBS between 35–37 weeks of pregnancy did not show the need for antibiotic administration. Their ages (range, mean age ± SD) and the number of blood samples collected are shown in Fig 1. Blood samples were collected from pregnant women at planned antenatal visits in each trimester and up to 48 hour after delivery.

A healthy pregnancy was confirmed by physical and ultrasound examinations, and laboratory investigations in all pregnant subjects. All were non-smokers and did not receive any anti-inflammatory treatment.

The non-pregnant women were recruited among hospital staff based on a clinical interview. All were nonsmokers and had not used hormonal contraception for at least six months prior to blood sample collection.

Infections and any other health problems were the exclusion criteria for participating in the study for both pregnant and non-pregnant subjects.

### Blood samples

Blood samples were drawn using venipuncture into blood collection tubes which did not contain an anticoagulant and allowed to clot at room temperature. After centrifugation (at 3000 rpm) for 10 min at 4°C, the sera were portioned and immediately stored at -80°C until assayed. On the day of measurements, blood samples were thawed at room temperature using gentle vortexing.

### Measurements of albumin and AGP serum levels

Measurements of protein levels were performed using the colorimetric BCG **(Bromocresol Green)** method for Alb and the immunoturbidimetric method for AGP on the Cobas c502 analyzer (Roche Diagnostics, Basel, Switzerland), with original reagents (ALB2, AAGP2), calibrators and controls.

### Measurements of IS

#### Sample preparation

Each sample (100 μL of serum) was mixed with 200 μL of deuterated indoxyl sulfate (ISD4) solution in acetone (1 μg/mL), vortexed for 1 min and centrifuged for 2 min with the 17500 Relative Centrifugal Force (RCF). The supernatant was transferred into chromatographic vials for analysis. Eight calibration points were prepared (range: 100 ng/mL to 3000 ng/mL) and three quality control (QC) points: QC_1_ 300 ng/mL, QC_2_ 800 ng/mL and QC_3_ 2200 ng/mL.

#### Sample analysis

The samples were analyzed using the Waters Xevo TQ-S triple quadrupole mass spectrometer (parameters: Polarity ESI, Capillary (kV) 2.6) coupled to Waters Acquity I-Class Ultra Performance Liquid Chromatography (UPLC). A 2 min High-Performance Liquid Chromatography (HPLC) gradient method was set up on the Waters UPLC BEH 1.7 μm 2.1 × 100 mm column with thermostatic control at 70°C. Mobile phase A consisted of 2 mM ammonium acetate with 0.1% formic acid (v/v) in MQ water. Mobile phase B consisted of 2 mM ammonium acetate with 0.1% formic acid (v/v) in LC/MS grade ACN (acetonitrile). The injection volume was 15 μL and flow rate 400 μL/min. The gradient used for separation was changed in time (min): initial, 0.2, 1.0, 1.5 and mobile phases were respectively A (%) 90, 10, 2, 90 and B (%) 10, 90, 98, 10. Monitored transmissions were: 212.00 > 79.96 CE (Collision energy) 20, 212.00 >132.04 CE 20 –for indoxyl sulfate, and 216.11 > 135.76 CE 20 for indoxyl sulfate -D4.

The samples were blinded to the case-control status and the principles of Good Laboratory Practice were followed.

### Statistical analysis

Statistical analyses were performed using STATISTICA [StatSoft Inc. (2014) STATISTICA (data analysis software system) version 12. www.statsoft.com]. The results are expressed as mean ± standard deviation (SD), median, and range. The Shapiro-Wilk test was performed to assess the normality of distribution. The Anova rang Kruskal-Wallis test and the Spearman’s rank order test were performed to compare the levels and correlations, respectively. The results were considered statistically significant when P value was < 0.05.

## Results

**Fig 2:**
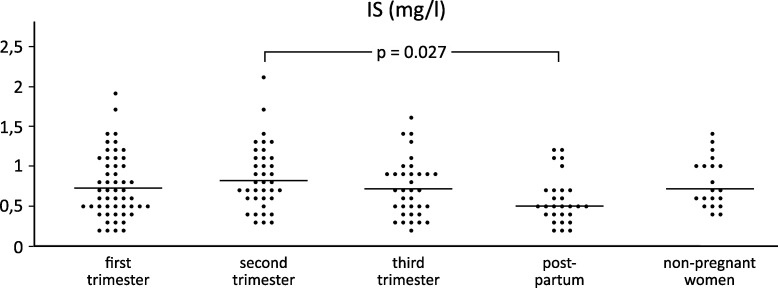
The scatter of individual serum IS levels in the first, second and third trimesters, in the postpartum period, and in non-pregnant women. Vertically aligned dots represent individual levels in the above study groups. Horizontal lines represent median serum IS levels.

**Fig 3:**
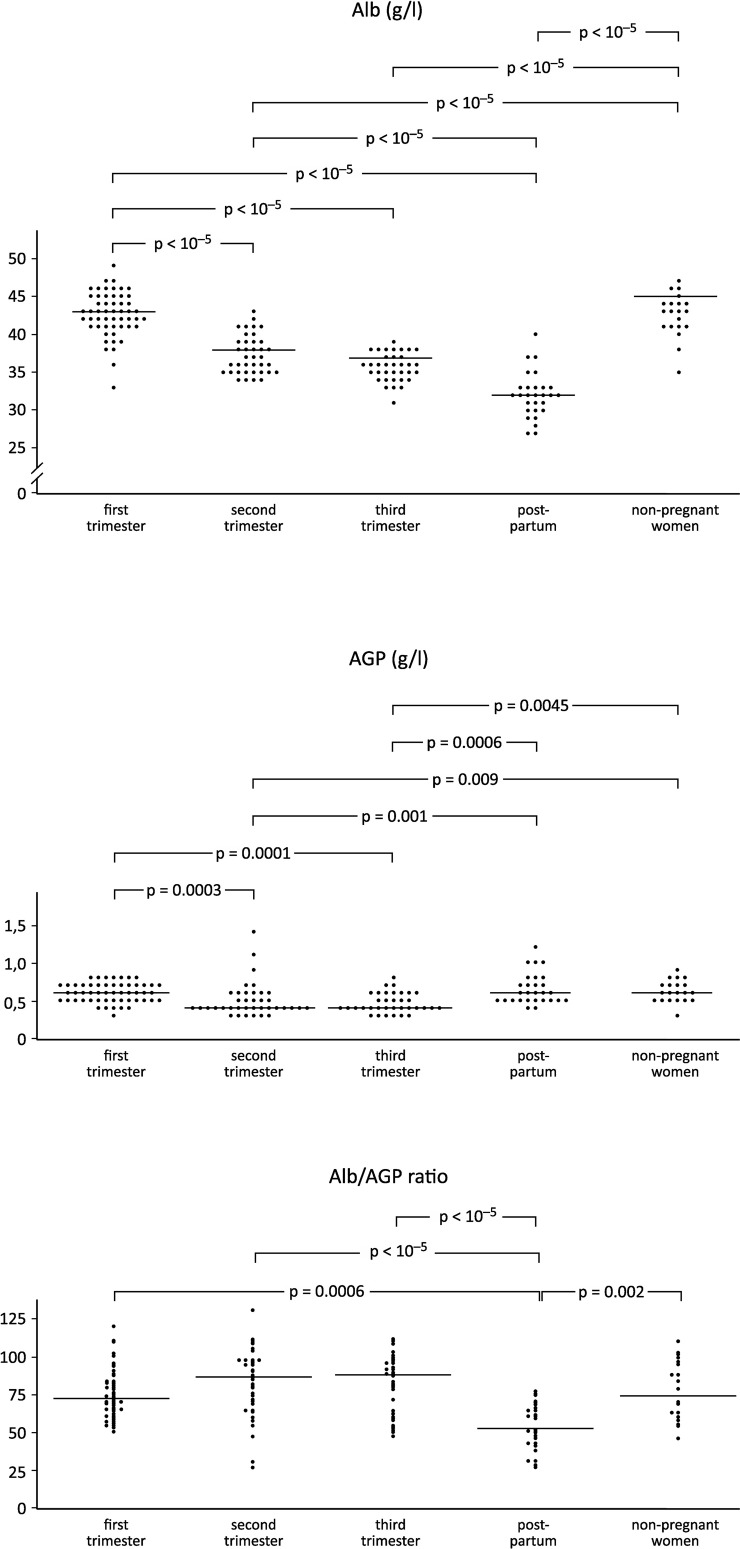
The scatter of individual serum Alb and AGP levels in the first, second and third trimesters, in the postpartum period, and in non-pregnant women. Vertically aligned dots represent individual levels in the above study groups. Horizontal lines represent median serum IS levels.

Table 1 presents changes in the serum levels of IS, Alb and AGP in the three consecutive trimesters of pregnancy and the postpartum period, and in the non-pregnant controls. The distribution of the levels of IS, Alb and AGP and statistically significant differences between the trimesters of pregnancy and the postpartum period as compared with the non-pregnant controls was graphically evaluated (Figs 2 and 3).

**Table 1:** Serum levels of IS, Alb and AGP in the three trimesters of normal pregnancy and the postpartum period, and in non-pregnant women.

Parameter	Study groups $\bar{x}$ ± SD, *median*, (range)					Anova rank Kruskal-Wallis test
	Trimesters of pregnancy			Postpartum n = 28	Non-pregnant n = 20	
	First n = 53	Second n = 38	Third n = 35			
IS (mg/L)	0.75 ± 0.39	0.85 ± 0.39	0.72 ± 0.36	*0.58 ± 0.32	0.80 ± 0.32	p = 0.038
	0.68 (0.16–1.87)	0.79 (0.30–2.05)	0.70 (0.21–1.63)	0.51 (0.16–1.31)	0.73 (0.35–1.45)	
Alb (g/L)	42.6 ± 2.9	**37.3 ± 2.5	**35. 7 ± 1.8	***32.0 ± 2.9	44.6 ± 2.8	p < 10^−4^
	42.5 (33.2–48.5)	36.8 (34.0–42.5)	35. 5 (31.3–38.8)	32.0 (27.0–40.0)	45.0 (37.0–49.0)	
AGP (g/L)	0.59 ± 0.12	**0.49 ± 0.22	**0.46 ± 0.12	***0.64 ± 0.2	0.61 ± 0.14	p < 10^−3^
	0.62 (0.3–0.81)	0.43 (0.31–1.36)	0.42 (0.31–0.77)	0.56 (0.4–1.19)	0.61 (0.32–0.91)	

$\bar{x}$ - mean, SD–standard deviation.

*—a significant decrease in serum IS (p = 0.027) compared with the second trimester.

**—a significant decrease in serum Alb (p<10−^5^) and AGP (p = 0.0003) compared with the first trimester and the non-pregnant state.

***- a significant decrease in serum Alb (p<10−^5^) and an increase in serum AGP compared with the second and third trimesters of pregnancy.

The serum IS levels did not change in the three trimesters of pregnancy and did not differ from the measurements in the non-pregnant controls. In the postpartum period, the IS levels slightly decreased (p = 0.027) compared to the second trimester.

The mean levels of serum Alb and AGP tended to decrease with progression of pregnancy, although at a different rate. Alb gradually decreased from trimester to trimester, was lower than in non-pregnant controls and further declined in the postpartum period. AGP was lower in the second and third trimester than in the first trimester and in non-pregnant controls. Unlike Alb, AGP significantly increased in the postpartum period compared to the second and third trimesters.

Table 2 shows changes in the IS to Alb ratio, IS to AGP ratio, and Alb to AGP ratio in the three trimesters of pregnancy and in the postpartum period compared to the measurements in non-pregnant controls.

**Table 2:** The relationship between serum IS, Alb and AGP levels in the three trimesters of normal pregnancy and the postpartum, and in non-pregnant women.

Ratio	Study groups $\bar{x}$ ± SD *median* (range)					Anova ran Kruskal-Wallis test
	Trimesters of pregnancy			Postpartum n = 28	Non-pregnant n = 20	
	First n = 53	Second n = 38	Third n = 35			
Alb/AGP	75 ± 17	84 ± 23	82 ± 19	*54 ± 15	78 ± 19	p < 10^−4^
	***73*** (51–120)	***8****6* (26–133)	***88*** (46–113)	***52*** (27–77)	***75*** (46–116)	
IS/Alb (μg/g)	17.8 ± 9.6	22.9 ± 10.6	20.2 ± 10.0	18.4 ± 10.0	17.9 ± 6.6	p = 0.151
	***15*.*0*** (4.0–42.0)	***22*.*5*** (8.0–54.0)	***20*.*0*** (6.0–45.0)	***15*.*5*** (5.0–41.0)	***15*.*5*** (9.0–33.0)	
IS/AGP (mg/g)	1.34 ± 0.85	**1.91 ± 1.11	1.66 ± 0.93	***0.97 ± 0.58	1.33 ± 0.50	p = 0.0003
	***1*.*20*** (0.3–3.8)	***1*.*75*** (0.5–5.0)	***1*.*50*** (0.5–4.4)	***0*.*90*** (0.2–3.1)	***1*.*25*** (0.7–2.4)	

$\bar{x}$ - mean, SD–standard deviation.

* a significant decrease in Alb to AGP ratio in the postpartum period compared with the first, second and third trimester (p = 0.0006, p<10^−5^, p<10^−5^, respectively) and in the non-pregnant state (p = 0.002).

** a significant increase in the IS to AGP ratio from the first to the second trimester (p = 0.039).

*** a significant decrease in the IS to AGP ratio in the postpartum period compared with the second and third trimester (p = 0.0003, p = 0.01, respectively).

The dynamics of changes in serum Alb/AGP, IS/Alb and IS/AGP ratios in pregnant women differs between the trimesters of pregnancy and in the postpartum period. The mean serum level of Alb was nearly 100-fold higher than that of AGP. The Alb to AGP ratios in the three trimesters of pregnancy did not differ from the ratios calculated for the controls, but they were significantly lower in the postpartum period than in any trimester of pregnancy and in the non-pregnant state. There were no differences in the IS /Alb ratio between the trimesters of pregnancy, the postpartum period and the non-pregnant controls. Similarly, the IS/AGP ratio during pregnancy did not differ from non-pregnant controls, while the IS/AGP ratio demonstrated characteristic alterations along pregnancy, with an increase from the first to the second trimester and a decrease in the postpartum period compared to the second and third trimester.

## Discussion

Our results confirm the relationship between serum levels of IS and AGP in healthy women during pregnancy and in the postpartum period and suggest the potential use of these laboratory parameters as biomarkers of intestinal homeostasis during pregnancy and in the postpartum period.

Although IS and AGP are extensively studied and well-characterized components of human serum, to date little is known about their possible use as gut-specific diagnostic parameters. Increased levels of IS and its accumulation in the serum result from factors contributing to its biosynthesis (dietary tryptophan, activity of intestinal gram-positive and gram-negative bacteria tryptophanase which converts tryptophan to indole, indole absorption into the systemic circulation and its conjugation with sulfate in the liver) [12, 13] and the rate of IS removal from urine via clearance by tubular secretion in the kidneys [2, 11]. At present, most studies focus on the toxicity of increased serum levels of IS and their role in the progression of renal disease and vascular disease, and the adverse effects on bones and the central nervous system [4, 9, 10]. Most of literature data concerning the properties of IS comes from experimental studies in cultured cells and animal models, while the present study was performed in a group of healthy pregnant women [4, 8]. No significant increases in the serum IS levels were found in healthy pregnancy compared to the non-pregnant state and there were no differences in the IS levels between the trimesters of pregnancy. This finding rules out the effect of renal function and suggests an equilibrium between the mechanisms regulating IS biosynthesis and its clearance from the body. An interesting exception is the slight decrease in serum IS levels in the postpartum period compared with those measured during pregnancy. Based on literature data, this finding could be accounted for by the dramatic postpartum hormonal changes, with impact on the composition and concentration of gut microbiota [20, 21], resulting in altered levels of both indole produced in the intestinal lumen and IS in maternal serum.

With decreased levels of progesterone and estrogen in the postpartum period, a decrease in IS serum levels may signal an increased risk of adverse biological effects due to the altered intestinal barrier function. Recent evidence suggests that microbial alterations seen during pregnancy may help maintain homeostasis and aid the required physiological changes occurring in pregnancy. Experimental studies have emphasized the importance of indole synthesized in the colon for enhancing the epithelial barrier function as it mediates the up-regulation of tight junction-associated molecules, controls diverse aspects of bacterial physiology, including intercellular signals in bacteria, and contributes to resistance to intestinal inflammation [7, 8]. There is limited information on gut microbial characteristics in pregnant women [22]. Clinical observations of beneficial effects of progesterone and estrogen in active inflammatory bowel disease and their anti-inflammatory and immunomodulatory effects on the intestinal epithelial lining during pregnancy until the third trimester suggest that they may be involved in improving the intestinal epithelial barrier functions [23, 24].

The question arises whether, as a result of a simultaneous reduction in the levels of serum sex hormones and gut indole in women after childbirth, there may be an increased migration of bacteria through the intestinal epithelium into the systemic circulation, increasing systemic inflammation. Consistently, selection of individual serum proteins as diagnostic tools specifically associated with altered IS levels may help understand the mechanisms underlying IS involvement in metabolic processes and assess potential uses of IS as a diagnostic marker.

The proportions of IS, Alb and AGP (the ratios of IS to Alb and IS to AGP) found in this study in the three trimesters of pregnancy and the postpartum period showed that the relationships between IS and Alb and of IS and AGP differ. Although most authors point to Alb as the key transport protein for IS [13], we did not demonstrate any significant differences in the IS to Alb ratio between the trimesters of pregnancy and the postpartum period. Characteristic changes in the IS to AGP ratio, with an increase in the second trimester and a drop in the postpartum, suggest some specific relationship between IS and AGP. AGP is generally recognized as an acute phase protein involved in immune responses. This abundant plasma protein is an immunomodulator induced by stressful conditions such as infections, and it protects adipose tissue from excessive inflammation induced by incresing plasma IS levels [15, 16, 25, 26]. The authors speculate that the serum AGP may specifically refer to the gut function as a highly reliable indicator of disease activity in ulcerative colitis and diarrhea-predominant irritable bowel syndrome [17, 27]. The increase in the IS to AGP ratio in the second trimester and its postpartum decrease may indirectly indicate the impact of hormonal changes on the alterations in the gut microbiota composition in two periods: during early prenatal life and postpartum [28, 29]. The postpartum drop in the IS to AGP ratio may reflect increased intestinal permeability. It seems to confirm other studies which reported in the postpartum period an increased risk for the penetration of bacteria and bacterial components like lipopolysaccharides which may alter susceptibility to infection and inflammatory disease in this period [30, 31]. Summing up, the changes in IS serum levels found in this study probably due to the hormonal and microbiota alterations and the parallel changes in serum AGP, a proinflammatory and immunoregulatory protein, may indicate their functional interrelationship. The selection of these two serum components may show a possible indirect linkage of intraintestinal production of IS.

## Supporting information

S1 Data (XLSX)
